# Ultrafine Metallic Particles as Inducers of Digestive Processes in Rumen: Dry Matter Digestibility of Feed and Enzymatic Activity

**DOI:** 10.1155/sci5/9556646

**Published:** 2025-08-30

**Authors:** Daniil E. Shoshin, Elena A. Sizova, Elena V. Yausheva, Kristina V. Ryazantseva, Kseniya S. Nechitajlo, Aina M. Kamirova

**Affiliations:** ^1^Department of Physiology, Biochemistry and Morphology of Animals, Federal Research Centre of Biological Systems and Agrotechnologies of the Russian Academy of Sciences, Orenburg 460000, Russia; ^2^Scientific and Educational Center “Biological Systems and Nanotechnologies”, Orenburg State University, Orenburg 460018, Russia

**Keywords:** ciliates, cobalt, digestibility, enzymes, manganese, nitrogen forms, rumen digestion, ultrafine particles

## Abstract

The ban on the use of antibiotics in animal husbandry encourages an active search for highly effective alternatives with additional properties, one of which is ultrafine particles (UFP) of metallic nature. The objective of the presented work was to conduct a comprehensive analysis of newly synthesized UFP Co_3_O_4_ and Mn_2_O_3_, including determination of their biological activity on the model of luminescent bacterial strain and potentiating effect on rumen digestion in ruminants using an in situ method. In parallel, the activity of proteinase, lipase, amylase, and cellulase, as well as nitrogen forms, microbial biomass, and the number of protozoa in 1 mL of rumen fluid, were determined. The minimum inhibitory concentrations for Mn_2_O_3_ and Co_3_O_4_ UFP were 3.9 × 10^−2^ and 1.2 × 10^−3^ mg/mL, respectively. The digestibility coefficient with the introduction of Mn_2_O_3_ UFP (39.0 mg/kg dry matter of feed) increased relative to the control by 6.6% (*p* = 0.012); Co_3_O_4_ UFP (0.6 mg/kg) by 12.7% (*p* = 0.012). Cellulolytic, amylolytic, and lipolytic activities in the group with Mn_2_O_3_ UFP increased by 18.2%, 515.5%, and 122.6% times compared to control, respectively. Proteinase activity decreased by 7.7% compared to control. Similar indicators in the group with Co_3_O_4_ UFP were +35.1%, +210.3%, +74.2, and +8.8%. Other indicators changed accordingly. Thus, UFP Mn_2_O_3_ and Co_3_O_4_ demonstrated significant potential as effectors of digestive processes in the rumen, stimulating the reproduction of protozoa and the enzymatic activity of the microbiome, which in combination ensured an increase in the digestibility of dry matter of feed. In other words, they can be used in the future as feed additives for ruminants. However, to fully understand the mechanisms of their action, it is also necessary to analyze the microbiome and metabolic pathways in the rumen.

## 1. Introduction

Due to their specific characteristics, namely, a significant surface area-to-volume ratio and as a consequence high-activity [1] ultrafine particles (UFPs), nanoparticles or complexes are increasingly used in various sectors of the national economy, including medicine [2], veterinary medicine [3], agriculture [4], and animal husbandry [5]. They are widely used for both therapeutic and clinical purposes in cancer theranostics [6], treatment of diabetes [7], pain syndrome [8], asthma [9], and allergies [10], in regenerative medicine for organ and tissue transplantation [11], as antimicrobial and antiviral drugs [12, 13], drug carriers [14], food additives with highly absorbable macro- and microelements [15], mineral fertilizers [16], pesticides [17], and herbicides [18]. The introduction of UFPs into animal husbandry and feeding of farm animals as an alternative to antibiotic drugs [19] is especially relevant against the background of widespread bans on the use of the latter for therapeutic purposes or as growth promoters [20], which is largely due to the risks of the total spread of microorganism resistance to a wide range of drugs [21, 22] and the high probability of antibiotic accumulation in food products [23, 24].

Thus, in animal nutrition, UFPs are mainly used as an alternative to antibiotic preparations, as well as a source of macro- and microelements, which allows not only to increase their bioavailability but also to avoid competition for membrane transporters in the intestinal epithelial lining, reduce excretion and environmental pollution, while improving digestive processes, immunity, and productivity of livestock and poultry [25]. Thus, studies on artificial rumen have shown that the introduction of UFPs Zn (100 and 200 mg/kg) into feed leads to an increase in the concentration of volatile fatty acids, microbial protein, and the coefficient of digestibility of organic matter at the 6th and 12th hours of the incubation period [26]. In similar experiments with lower doses (20 mg/kg), a decrease in methane emanation was noted [27]. The same effects are projected in vivo: In sheep, the addition of UFPs Zn to the diet significantly increases the digestibility of dry and organic matter, nitrogen, and nitrogen-free extractives [28]. There was also a positive effect in response to dietary supplements with 50 mg UFPs of ZnO per 1 kg of dry matter (DM) on the digestibility of diet components in Holstein calves [29]. UFPs ZnO, as well as Zn-methionine, increased egg production and egg weight in laying hens (*p* < 0.05). In addition, in the UFPs ZnO group, the thickness and strength of the shell increased, the activity of superoxide dismutase (SOD) in the liver, pancreas, and blood plasma increased, and the concentration of malonic dialdehyde (MDA) in eggs decreased. In parallel, such blood parameters as total serum protein, albumin, glucose, alkaline phosphatase, and carbonic anhydrase activity, and Zn levels changed [30].

Male goats receiving 0.03 mg/kg UFPs Se had a higher final body weight and average daily weight gain than those receiving sodium selenite or Se yeast [31, 32]. In sheep, the addition of UFPs Se at a dose of 3 mg/kg to the basic diet significantly reduced the ruminal pH (6.68–6.80) and ammonia concentration (9.95–12.49 mg/100 mL) and linearly increased the total concentration of volatile fatty acids (range 73.63–77.72 mM) [33]. In another study on sheep, UFPs Se at a dose of 1 mg/kg reduced peroxide damage to blood components [34]. The addition of UFPs Se to the diet of newborn lambs contributed to an increase in the level of SOD while reducing the values of reactive substances of thiobarbituric acid (TBARS) [35].

Similarly, UFPs Se (0.9 mg/kg) improved intestinal health by increasing the number of beneficial bacteria, such as *Lactobacillus* and *Faecalibacterium*, and short-chain fatty acids, in particular butyric acid, but did not significantly affect body weight gain or the number of potentially pathogenic bacteria [36].

The introduction of Cu UFPs into the diet of Holstein heifers increased the growth rate, stimulated the function of hematopoiesis, and improved the characteristics of mineral metabolism [37]. At the same time, the addition of UFPs Cu to the feed of black-and-white calves at a dose of 0.04 mg/kg of live weight per day increased the digestibility of DM by 2.3%, organic matter by 4.1%, protein by 2.5%, fat by 0.5%, fiber by 3.1%, nitrogen-free extractive substances by 2.9% compared with the control, and the deposition of nitrogen in the animal body increased by 10.4%, calcium—by 14.2% and phosphorus—by 8.7% above the control. Similarly, the Fe UFPs increased the average daily increments by 67.2%. The monthly gross gains of the experimental animals increased by the same amount, the mass of the paired carcass increased by 43.9%, the carcass yield by 7.2%, the slaughter weight by 43.5%, and the slaughter yield by 8.7% compared with the control. The protein content in meat increased by 2.9%, essential amino acids by 3.75%, and ash by 0.3%, including cobalt by 3.15%, copper by 1.9%. The content of fatty acids in the subcutaneous fat of the experimental animals did not differ from the control values; therefore, UFPs did not cause pathological changes in lipid metabolism. It is also noted that the slaughter products fully comply with all the norms of San PIN 2.3.2.1078–01, and the structure and development of tissues and organs are within the physiological norm [38].

The introduction of UFPs Cu improved the blood profile and the mass index of lymphoid organs in broilers compared with the control group and the group receiving CuSO_4_. At the same time, the erythrocyte sedimentation rate, the ratio of neutrophils and lymphocytes, the concentration of malondialdehyde (MDA), and the hormone corticosterone decreased. In addition, there was a positive effect of UFPs Cu on all behavioral patterns, which significantly affects body weight, average daily gains, and feed consumption [39].

In piglets, the addition of UFPs Cu in the amount of 50 mg/kg led to a significant improvement in growth indicators. The level of copper in the stool was reduced, and the availability of copper was significantly improved compared to the group receiving regular copper sulfate (CuSO_4_). Significant differences were also observed in improving the digestibility of crude fat and energy in pigs fed a diet with UFPs Cu. Statistically significant improvements were observed in the levels of IgG protein, Y-globulin, and total globulin, as well as in SOD activity [40].

Dietary supplement UFPs Cr at the rate of 200 mg/kg significantly reduced the levels of glucose, urea nitrogen, triglycerides, cholesterol, and nonesterified fatty acid in the blood serum of weaned pigs. On the contrary, the level of total protein, high-density lipoproteins, and lipase activity in the blood serum increased significantly. There was also an increase in serum insulin-like growth factor I and a significant decrease in insulin and cortisol levels. In addition, additional intake of UFPs Cr led to an increase in the level of immunoglobulins, in particular IgM and IgG in plasma [41]. UFPs Cr also had a noticeable effect on carcass characteristics, pork quality, skeletal muscle mass, and increased Cr concentration in individual muscle tissues and organs [42].

The addition of UFPs Ag in amounts of 20 and 40 mg/kg as an antimicrobial agent and growth stimulator during the transition phase to the diet of weaned piglets (weight 5–20 kg) led to a decrease in the number of coliform bacteria in the intestinal contents. In addition, the concentration of the pathogen *Clostridium perfringens* or *Cl. histolyticum* group in the ileum was reduced with the use of 20 mg/kg of silver [43].

UFPs Ag reduced the number of *E. coli, Streptococcus,* and *Salmonella* bacteria and the total number of mesophilic bacteria in the litter [44]. Studies have also shown that nanosilver as a feed additive had a positive selective effect on the number of bacteria in the digestive tract of poultry. When adding 20, 40, and 60 mg/kg of UFPs Ag to the feed, the weight of the lymphatic organs decreased dose-dependently [45]. The lowest weight was observed when feeding 60 mg/kg on the 42nd day of life. This weight loss correlates with the antimicrobial property of Ag, which can lead to a favorable ratio of nonpathogenic and pathogenic organisms in the intestine. The broiler diet enriched with silver nanoparticles reduced the hemoglobin level and the number of erythrocytes and leukocytes [46]. A study by Andi et al. [47] showed an improvement in feed intake, growth, and feeding efficiency of broilers, due to the effect of silver ions on harmful intestinal bacteria and improved intestinal health and, consequently, better absorption of nutrients.

Thus, the use of UFPs of copper, selenium, chromium, zinc, iron, cobalt, and others as mineral additives can improve animal productivity and has a number of advantages over inorganic salts and organic chelated minerals [48]. At the same time, UFPs most often act on representatives of the fauna kingdom not only directly but also indirectly through symbiont bacterial ecosystems, such as the ruminant rumen microbiome, shifting the ratio of individual taxa, and leveling the number of pathogenic strains [49].

In turn, the priority of using UFPs in animal husbandry is determined by the high efficiency of a small dose, better bioavailability, and stable interaction with other compounds [50, 51]. In addition, nanoadditives can be integrated with micelles, protein capsules, or any feed ingredient [52].

At the same time, however, some characteristics of UFP, such as high adhesion to the cell wall surface [53], active binding to biological substrates [54], low degradation rate and the resulting minimization of pressure from intestinal clearance, prolongation of residence time in the gastrointestinal tract of animals, rapid absorption by mucosal cells, and penetration into deep tissues through the capillary bed [55], act simultaneously as both positive and negative factors, prompting a balance between the necessary therapeutic or nutritional effect and metabolic overload, resulting in poisoning [56]. Moreover, the properties of UFPs are very labile and depend not only on the nature, chemical elements, or sizes included in their composition [57] but also on the synthesis routes [58] and on the presence of additional alloying components and relationships with the environment [59, 60], in particular, on the formation of the so-called “organic” or “protein” crown [54, 61].

In this regard, before using any synthesized UFPs in feeding farm animals, it is necessary to conduct a detailed assessment of their biological properties in vitro and in vivo. Thus, the purpose of the present work was a comprehensive analysis of the synthesized metallic UFPs Co_3_O_4_ and Mn_2_O_3_, including the determination of their biological activity on the model of a luminescent bacterial strain and the potentiating effect on ruminal digestion in ruminants using an in situ method.

## 2. Materials and Methods

The experiment was carried out in two stages (in vitro and in situ) at Orenburg State University (https://www.osu.ru/) and the Center for Collective Use of the Federal Research Centre of Biomedical Engineering of the Russian Academy of Sciences:

### 2.1. Sample Preparation

UFPs of Co_3_O_4_ and Mn_2_O_3_, manufactured in the Laboratory of Nanostructure Synthesis of the Orenburg State University (OSU, Russia), in an amount of 40 mg each (laboratory scales VLA, accuracy class I, permissible error ± 0.5 mg), which corresponds to 0.25 M of a pure metal element, were dispersed by ultrasound at a frequency of 35 kHz in 1 mL of distilled water (USW “Sapphire” 4.0, Russia) for 30 min at a temperature of 25°C. After that, a series of twofold dilutions of the obtained suspensions were prepared in 96-well culture plates for further analysis in an amount of 100 μL in each well. At the same time, suspensions of UFPs with concentrations of 20 mg/mL were prepared, after that their diameter and ζ-potential were determined as a measure of suspension stability using the dynamic light scattering (DLS) method on a Microtrac NANOTRAC WAVE II laser analyzer (OOO Microtrac, Russia).

### 2.2. Effective Concentrations of UFP

The biological activity of UFP was assessed using a constitutively luminescent strain of *Escherichia coli K12 TG1* (commercial name “Ekolum,” “NVO IMMUNOTECH,” Russia) carrying a hybrid plasmid *pUC19* with cloned *luxCDABE* genes of *Photobacterium leiognathi 54D10* on a multifunctional microplate reader TECAN Infinite F200 (Tecan Austria GmbH, Austria). For this purpose, 5 mL of water cooled to 4°C and at room temperature was added to the initial lyophilisate, vigorously shaken until the sediment was completely dissolved, and kept in the refrigerator for 30 min. After that, 100 μL of the resulting suspension was added to the prepared dilutions of UFP, obtaining final concentrations from 20 to 2.4 × 10^−6^ mg/mL (hereinafter in terms of pure metal elements). The plate with experimental samples was placed in the device, recording the luminescence intensity in relative instrumental units (relative luminescence units, RLU) every 5 min for 3 h of exposure. For graphic display of the obtained results, the toxicity index was calculated using the following formula:

(1) $$\begin{array}{c} Τ=\frac{I_{k}-I_{o}}{I_{k}}\times 100\%, \end{array}$$

where *I*_*k*_—luminance of control sample, RLU, and *I*_*o*_—luminance of experimental sample, RLU.

At the same time, effective concentrations suppressing 80% (EC_80_), 50% (EC_50_), and 20% (EC_20_) of luminescence were determined, i.e., toxic, conditionally toxic, and subinhibitory doses, respectively. The latter were recommended for further testing on animals.

The effectiveness of the method was proven using the example of *Vibrio fischeri* and *Escherichia coli* strains in the analysis of wastewater toxicity [62] and the bactericidal activity of ultrafine metal particles [63, 64].

### 2.3. In Situ Digestibility of Feed DM

The digestibility of DM of the base substrate (wheat bran) with the addition of the studied UFPs was determined using the nylon bag method [65]. For this purpose, nylon bags with 100 g of wheat bran mixed with UFP Co_3_O_4_ (0.6 mg/kg feed DM, Experiment 1), UFP Mn_2_O_3_ (39,0 mg/kg feed DM, Experiment 2), and without additives (Control) were placed into the rumen of Kazakh White-headed bulls (*n* = 5, average weight 266 ± 1.53 kg, aged 11–12 months), their diet included cereal hay (1 kg), legume hay (2 kg), corn silage (9.5 kg), crushed grain mixture (2 kg), sunflower cake (0.1 kg), feed molasses (0.6 kg), table salt (37 g), monocalcium phosphate (47.7 g), and premix (20 g) through a chronic fistula (*d* = 80 mm, ANKOM Technology Corporation, USA). Experimental dosages of UFPs were selected based on the experience described above in determining effective concentrations. Subinhibitory dosages suppressing 20% of the luminescence of *Escherichia coli K12 TG1* (EC_20_) were allowed for animal testing.

At the end of the incubation, the samples were washed and dried at a temperature of +100°C to a constant weight (Drying cabinet GP-80 SPU, JSC Smolensk SKTB SPU, Russia). The digestibility coefficient was determined by the following formula:

(2) $$\begin{array}{c} K=\frac{100-\left(m_{1}-m_{2}\right)}{100}\times 100\%, \end{array}$$

where *m*_1_—weight of dried bags of feed after digestion, *g*; *m*_2_—weight of bags without feed, *g*.

All the studies described below were performed according to the methods proposed by Senko AV and Voronov DV (2010) [66].

### 2.4. Protozoa Count and Microbial Biomass

After 24 h of digestion, rumen contents were sampled. The samples were transported for 30 min, maintaining a temperature of +38.5°C …+39.5°C. Before the study, ruminal fluid was thoroughly shaken and filtered through 4 layers of gauze. The protozoa count in ruminal fluid was determined using a Goryaev chamber. For this purpose, 5 mL of the filtered rumen contents was collected in a test tube and 0.1 mL of a 4% formalin solution was added to fix the ciliates and 20 μL of methylene blue. The mixture was shaken for 1–2 min. One drop of liquid was added to the chamber with a Goryaev grid under a cover glass and the number of ciliates was counted in 25 large squares, after which the number of protozoa in 1 mL of rumen contents was determined using the following formula:

(3) $$\begin{array}{c} N=\frac{1000\times n\times b}{S\times h}=\frac{1000\times n\times 2}{25\times 0,04\times 0,1}=20000n, \end{array}$$

where *n*—the number of cells counted in a given sector; *b*—sample dilution factor; S—area of the sector under study; and *h*—counting chamber depth.

The total microbial mass was determined by centrifugation and threefold to fivefold washing at 10,000 g for 15 min (Mini centrifuge, GYROZEN Co., Ltd., South Korea).

### 2.5. Enzymatic Activity of Rumen Contents and Nitrogen Forms

1. Cellulolytic activity of ruminal fluid microflora. 10 mL of freshly taken rumen contents and 0.3 mL of 16% glucose solution were mixed in a test tube, preweighed (≈100 mg) defatted cotton thread was added, filled with Vaseline oil, and tightly closed with a rubber stopper. Then, the test tube was placed in a TS-1/80 SPU thermostat (OJSC Smolensk SKTB SPU, Russia) at 40°C for 48 h, after which the thread was taken out, washed, and dried to a constant weight, recording the change in weight. The degree of cellulose digestibility was expressed in mg/h per 1 mL of ruminal fluid:
  (4) $$\begin{array}{c} Aс=\frac{\left(m_{1}-m_{2}\right)}{48\times 10}, \end{array}$$
where *m*_1_—weight of thread before digestion, mg; *m*_2_—weight of thread after digestion, mg; 48—exposure time, h; and 10—conversion factor for 1 mL of rumen fluid
2. Amylolytic activity of ruminal fluid microflora. In a test tube, 1.6 mL of 6.25% starch substrate solution was mixed with 7.4 mL of phosphate buffer (pH 6.8). After heating the test tube for 10 min in a LOIP LB-160 water bath (JSC Laboratory Equipment and Devices) at 40°C, 1 mL of ruminal fluid filtered through 2 layers of gauze was added. The contents of the test tube were thoroughly shaken, and 0.5 mL of this liquid was immediately transferred with a pipette into a 50-mL measuring flask with 2 mL of 2 N HCl solution to stop the action of microbial enzymes. Two mL of potassium iodide solution was added to the flask, and the volume was brought to 50 mL with distilled water (sample before incubation). After this, the test tube was kept for 1 h in a water bath at a temperature of 40°C, periodically (every 10–15 min) mixing the contents of the test tube by shaking. Upon completion of the incubation, a 0.5 mL of the sample was taken from the test tube and transferred to a 50-mL measuring flask with 2 mL of a 2 N HCl solution. Two mL of potassium iodide and distilled water was added to the mark (sample after incubation). The resulting sample solutions before incubation and 1 h after it were examined on an SF-2000 spectrophotometer (OKB SPEKTR, Russia) in 10-mm cuvettes with a red-light filter (620 nm) against distilled water.
The calibration curve was used to determine the amount of starch (mg) in the solutions before and after incubation. The difference between these values gives the amount of starch broken down by microbial amylase during 1 h of incubation. The degree of starch digestibility was expressed in mg/h per 1 mL of ruminal fluid:
  (5) $$\begin{array}{c} Αa=\left(m_{1}-m_{2}\right)\times 20, \end{array}$$
where *m*_1⁣_—weight of starch in solution before incubation, mg; *m*_2_—weight of starch in the solution after incubation, mg; and 20—conversion factor for 1 mL of ruminal fluid;
3. Proteinase activity of ruminal fluid microflora. Four mL of casein solution and 1 mL of phosphate buffer (pH 7.1) were added to one test tube (experiment) and placed in a water bath at 40°C. After 10 min, 1 mL of rumen contents was diluted 5 times with distilled water and incubated at 40°C for 60 min. Then, 4 mL of 10% trichloroacetic acid solution was added to stop the reaction. The mixture was shaken and centrifuged at 1500 rpm (Centrifuge SM-12, Techno-Com, Russia) for 10 min (the supernatant should be transparent). A 1-mL aliquot was collected, 19 mL of solution “C” was added (100 mL of 1% ninhydrin solution in citrate buffer (pH 5.4) + 40 mL of 60% glycerol + 40 mL of citrate buffer), and the tubes were sealed with ground glass stoppers and placed on a magnetic stirrer with heating MSH-300 (SIA Biosan, Latvia) at 120°C and boiled for 20 min (at pH greater than 5.0, OC–amino acids react with ninhydrin to form carbon dioxide, aldehyde, and a blue-colored compound, and the color intensity is directly proportional to the amount of amino acids). The blue-colored samples were cooled in running water and photometrically measured after 10–15 min at a wavelength of 620 nm in a 10-mm cuvette.
One mL of diluted rumen contents, 1 mL of phosphate buffer, and 4 mL of trichloroacetic acid were added to the control test tube, and the mixture was kept for 10 min in a water bath at 40°C. After that, 4 mL of casein solution was added, mixed, and precipitated. Then, it was studied in the same way as the experimental sample.
The amount of free amine nitrogen was determined using a calibration graph. To plot it, previously prepared glycine solutions were taken and a series of dilutions were prepared that contained different amounts of this amino acid (μmol), and then, the reaction was carried out in the same way as described above. The enzyme activity was expressed in micromoles of formed amine nitrogen in 1 h per 1 mL of rumen contents:
  (6) $$\begin{array}{c} Ap=\left(m_{1}-m_{2}\right)\times 50, \end{array}$$
where *m*_1⁣_—amount of amino nitrogen in solution before incubation, μmol; *m*_2_—amount of amino nitrogen in solution after incubation, μmol; and 50—conversion factor for 1 mL of ruminal fluid.
4. Lipolytic activity of ruminal fluid microflora. The rumen contents were thoroughly mixed and diluted 1:9. A substrate mixture was prepared: 3 mL of sunflower oil, 3 mL of Ringer-Locke solution, 1 mL of 0.6% ascorbic acid solution, and 30 mg of bile. Three mL of the substrate mixture and 1 mL of the rumen contents were collected in 10-mL test tubes (1 test and 1 control) with ground glass stoppers. The test samples were placed in a thermostat at 40°C for 5 h, and the control ones were placed in a refrigerator. The samples were shaken for 5 min every hour. After 5 h, 60 μL of 20% phosphotungstic acid solution and 40 μL of Tashiro indicator were added to the test tubes. The test and control samples were titrated with 0.01 N NaOH solution until the pink color disappeared. Lipolytic activity was expressed in μmoles of free fatty acids formed during the interaction of the enzyme with the substrate in 1 h per 1 mL of ruminal fluid:
  (7) $$\begin{array}{c} Al=\frac{\left(V_{1}-V_{2}\right)\times 0,01\times 1000}{5}\times 10, \end{array}$$
where *V*_1_—volume of NaOH used for titration of the sample with inactivated enzyme, ml; *V*_2_—volume of NaOH used for blank titration, ml; 0,01—normality of NaOH titrant used; 1000—conversion factor for mol/L to μmol/mL; 5—exposure time, *h*; and 10—conversion factor for 1 mL of ruminal fluid.

Total and residual nitrogen were determined using the Kjeldahl method, protein nitrogen was determined by calculation based on the difference between total and residual nitrogen, and ammonia was determined using the microdiffusion method in Conway dishes.

### 2.6. Statistical Processing

The experimental data were processed using the software package Statistica 12 (Stat Soft Inc., USA) and Microsoft Excel (Microsoft, USA). The mean (M), standard deviation (±*σ*), standard error (±SE), and Spearman's rank correlation coefficient (*r*) were calculated. A nonparametric analysis method was used to compare the variants. Differences were considered statistically significant at ^∗^*p* ≤ 0.05 and ^∗∗^*p* ≤ 0.01.

## 3. Results

### 3.1. Effective Concentrations of UFP

**Figure 1:**
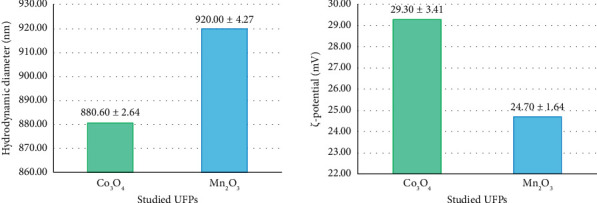
Hydrodynamic diameter and ζ-potential of the studied UFPs.

The hydrodynamic diameter of UFP Mn_2_O_3_ and Co_3_O_4_, determined by the DLS method, was 920.0 ± 4.3 and 880.6 ± 2.6 nm, respectively, at a ξ-potential of 29.3 ± 3.4 and 24.7 ± 1.6 mV (Figure 1).

Bactericidal activity increased with increased concentration. The zone of metabolic transition from indifferent to toxic parameters for UFP Mn_2_O_3_ was in the range from 2.0 × 10^−2^ to 10 (Table 1) and for UFP Co_3_O_4_—from 0.8 × 10^−4^ to 39.1 × 10^−3^ mg/mL (Table 2), respectively. The first demonstrated a pronounced bacteriostatic effect (the ratio of the RLU value at the end of the experiment to the same value in the first minute ranged from 88.82% to 120.12% depending on the concentration) and lower toxicity (inhibitory values were not revealed). Concentrations of UFP Mn_2_O_3_ from 20 to 5 mg/mL suppressed over 80%, from 2.5 to 0.16—over 50%, and from 7.8 × 10^−2^ to 3.9 × 10^−2^—over 20% luminescence. UFP Mn_2_O_3_ at a dose of 4.9 × 10^−4^ stimulated the luminescence of *Escherichia coli K12 TG1* in the range from 3.74% to 14.04% relative to the control.

When *Escherichia coli K12 TG1* suspension was contaminated with Co_3_O_4_ UFP in the range from 20 to 4.9 × 10^−3^ mg/mL, absolute suppression of luminescence was observed in the last minutes of exposure, while the luminosity of bacterial strain at the beginning of the experiment was the higher, the less Co_3_O_4_ UFP was introduced into the medium, and the toxicity index here varied from 49.45% to 99.70% as the concentration increased, respectively. Co_3_O_4_ UFP concentrations from 20 to 4.9 × 10^−3^ mg/mL suppressed over 80%; 2.4 × 10^−3^—over 50%; and 1.2 × 10^−3^—over 20% of luminescence. Furthermore, at 3.1 × 10^−4^ and 1.5 × 10^−4^ mg/mL of UFP Co_3_O_4_, a short-term increase in bioluminescence by 10.71% and 27.83% was detected, and in the first case, this effect lasted no more than 30 min, and in the second, 120 min.

Consequently, UFP Mn_2_O_3_ is 4000 times less toxic than UFP Co_3_O_4_ and is characterized by a significantly wider biochemical transition zone—9 twofold dilutions versus 3.

### 3.2. In Situ Digestibility of Feed DM

**Figure 2:**
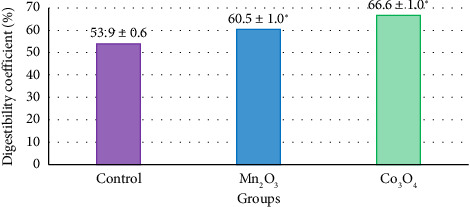
In situ digestibility of feed dry matter with the addition of the studied UFPs, 24 h of exposure, *n* = 5. *Note*: ^∗^*p* ≤ 0.05.

The digestibility coefficient of the base substrate with the addition of UFP Mn_2_O_3_ in the amount of 39.0 mg/kg feed DM (the dosage was determined by the EC_20_ criterion based on the biotoxicity assessment based on the fact of equating 1 mL of the reaction medium to 1 g of feed DM) increased relative to the control by 6.6% (*p* = 0.012) (Figure 2). Similarly, UFP Co_3_O_4_ at a dose of 0.6 mg/kg feed DM intensified digestive processes in the rumen by 12.7% (*p* = 0.012).

### 3.3. The Number of Protozoa and Microbial Biomass

**Figure 3:**
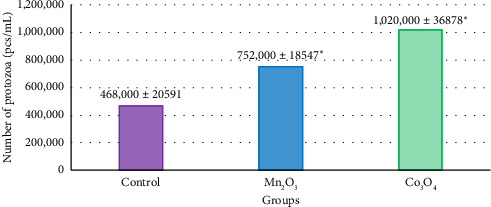
Number of protozoa in 1 mL of ruminal fluid in situ with the introduction of the studied UFPs, 24 h of exposure, *n* = 5. Note: ^∗^*p* ≤ 0.05.

**Figure 4:**
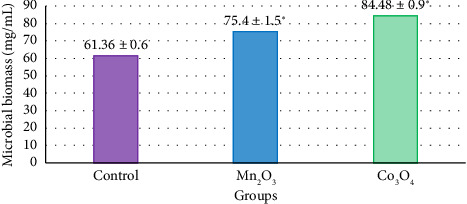
Microbial biomass per 1 mL of ruminal fluid in situ upon introduction of the studied UFPs, 24 h of exposure, *n* = 5. *Note*: ^∗^*p* ≤ 0.05.

In addition to the positive dynamics in the experimental groups (Figures 3 and 4), reliable correlations with the indicators of DM digestibility of feed were established regarding the number of ciliates in 1 mL of ruminal fluid and the total microbial biomass. Thus, the number of protozoa with the introduction of UFP Mn_2_O_3_ into the base substrate increased by 60.7% (*p* = 0.012), and the total microbial biomass—by 22.9% (*p* = 0.012) relative to the control, in the experiment with UFP Co_3_O_4_—by 117.9% (*p* = 0.012) and 37.7% (*p* = 0.012), respectively. At the same time, Spearman's rank correlation coefficient relative to digestibility for these two indicators was 0.89 (*p* < 0.001).

### 3.4. Enzymatic Activity

The introduction of the studied UFPs into the basic substrate was accompanied by an increase in the enzymatic activity of the microbiome (Table 3). Cellulolytic activity increased in the group with Mn_2_O_3_ UFP by 18.2% (*p* = 0.012); in the group with Co_3_O_4_ UFP—by 35.1% (*p* = 0.012); similarly, amylolytic activity increased more than 6 times in the first case, and 3 times in the second, lipolytic, respectively, by 2.2 and 1.7 times—in the second. Proteinase activity in the experiment with Mn_2_O_3_ UFP decreased by 7.7% (*p* = 0.022), but increased in the experiment with Co_3_O_4_ UFP—by 8.8% (*p* = 0.012).

Cellulolytic activity clearly correlated with digestibility indices (*r* = 0.86; *p* < 0.001), protozoa numbers (*r* = 0.89; *p* < 0.001), and bacterial biomass (*r* = 0.95; *p* < 0.001). The remaining indices tended to show this type of correlation with *r* = 0.38–0.50.

### 3.5. Nitrogen Forms

**Figure 5:**
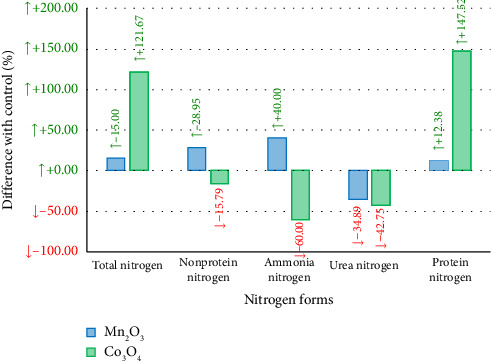
Dynamics of nitrogen forms relative to the control in ruminal fluid at the end of the experiment, *n* = 5. *Note*: ^∗^*p* ≤ 0.05.

The use of UFP Mn_2_O_3_ contributed to an increase in the concentration of total (*p* = 0.012), protein (*p* = 0.060), nonprotein (*p* = 0.012), and ammonia nitrogen (*p* = 0.012) in ruminal fluid (Figure 5), while the use of UFP Co_3_O_4_ led to a decrease in the last two parameters (*p* = 0.012). The amount of urea nitrogen decreased in both cases (*p* = 0.012).

It is also important to note that total nitrogen concentration was positively correlated with protozoan abundance (*r* = 0.83; *p* < 0.001), microbial biomass (*r* = 0.80; *p* < 0.001), digestibility (*r* = 0.87; *p* < 0.001), and proteinase activity (*r* = 0.61; *p* = 0.0014) and, similarly, for protein nitrogen: [*r* = 0.78, *p* < 0.001], [*r* = 0.75, *p* = 0.0014], [*r* = 0.83, *p* < 0.001], and [*r* = 0.67, *p* = 0.0061], respectively. Protein nitrogen was negatively correlated with ammonia (*r* = −0.64, *p* = 0.0097) and urea (*r* = −0.73, *p* = 0.0019) forms.

Thus, UFP Mn_2_O_3_ and Co_3_O_4_ demonstrated significant potential as effectors of rumen digestion in cattle, stimulating the reproduction of protozoa and the enzymatic activity of the microbiome, which in combination ensured an increase in the DM digestibility of feed.

## 4. Discussion

### 4.1. Bactericidal Properties of UFP

The bactericidal properties of UFP are mediated primarily by their small size and high active surface area, which constantly produces free ions. The latter, by binding to or penetrating cell membranes, induces the synthesis of reactive oxygen species (ROS), which is accompanied by disturbances in DNA repair, transcription and translation processes, and ultimately leads to cell apoptosis [67], and mitochondrial dysfunction and decreased ATP production are also possible [68]. At the same time, positively charged particles are the most toxic, easily crossing cellular barriers and binding to DNA [69]. In particular, regarding the UFP Co_3_O_4_, it has been established, for example, that chernozems contaminated with it are characterized by a decrease in the total number of bacteria, especially of the genus *Azotobacter*, the activity of catalase and dehydrogenases [70]. Similarly, after such contact, cell growth and synthesis of chlorophyll slows down in freshwater microalga *Chlorella minutissima* [71], with an effective concentration of EC_50_ = 38.16 ± 1.99 mg/L. Additionally, the possibility of using subtoxic concentrations of Co_3_O_4_ as an alternative to tetracycline and gentamicin drugs was shown in model organisms of *Escherichia coli* and *Staphylococcus aureus* [32] with effective doses of ≥ 125 μg/mL and ≥ 31.25 μg/mL, respectively [72].

With regard to Mn_2_O_3_ UFPs, it has been established that they reduce oxygen consumption in *Saccharomyces cerevisiae* by 20% at a dose of 50 mg/L and by 50% at a dose of 170 mg/L [73]. Mn_2_O_3_ nanowires have a bactericidal and cytotoxic effect, inhibiting the growth and reproduction of *Escherichia coli* and functional activity of mouse C_2_C_12_ myoblasts in an amount of 12.5 μg/mL [74]. Furthermore, one recent study showed that Mn_2_O_3_ nanoparticles with a diameter of 23 nm inhibit to varying degrees the growth and development of *Staphylococcus aureus*, *Enterococcus faecalis* (Minimum inhibitory concentration (MIC) = 2 μg/mL), *Escherichia coli, Proteus mirabilis* (MIC = 3 μg/mL), and *Klebsiella pneumoniae* (MIC = 3.5 μg/mL). The authors note that nanoparticles obtained through “green synthesis” are more toxic to gram-positive bacteria than to gram-negative bacteria, attributing this fact to the structure of the cell wall of the latter, which has an additional outer membrane with porin or porin-like proteins that mediate its permeability [75]. However, there is also an opposing point of view regarding this position. In particular, Azhir, E., et al. (2015) demonstrated that *Escherichia coli* is less resistant to Mn_2_O_3_ nanoparticles of similar size than *Staphylococcus aureus* [76]. In other words, the question of the mechanisms of toxicity of nanoparticles and UFPs still remains open, since the latter are determined by a whole complex of factors, including not only the size, shape, synthesis routes, type of material, and concentration, but also the model strains of microorganisms themselves, the composition of the physiological environment, and the presence of alloying components [77].

In this regard, the effective concentrations presented in our experiment somewhat diverge from those described in the cited works, which once again confirms the need for a preliminary assessment of each synthesized version of UFP. Nevertheless, Rana S. and Kumar A. (2022), studying a consortium of algae and bacteria, identify the following most general stages of UFP action: a) change in membrane permeability, surface adsorption, and damage to transport proteins; b) penetration of ions into the bacterial cell; c) interaction with cellular organelles; d) generation of ROS; and e) the onset of degenerative changes [78]. A similar effect is associated with the ability of variable valence metals to carry out lipid peroxidation processes and form substances that block the active centers of enzymes, including luciferase, which is responsible for bioluminescence. Moreover, the study of a complex of metal-containing particles ZnO, CuO, CoO, Mn_2_O_3_, Со_3_O_4_, Ni_2_O_3,_ and Cr_2_O_3_ showed that the behavior of UFP under identical characteristics and conditions both in relation to *Escherichia coli* and in the case of mammalian cell lines is very similar [79]. In this regard, as toxicity of UFP is significantly lower in comparison with nanoparticles, the certification of UFP on bacteria allows selecting optimal dosages that are harmless to the host macroorganism, but act simultaneously as an antibiotic drug and as a highly labile biocompatible source of microelements in nutrition—essential components of a wide range of accessory substances in the animal body, including enzymes [80]. Moreover, the advantages of UFP over inorganic salts are confirmed by a number of studies using UFP zinc [81], copper [82], iron [83], and cobalt in particular [84], which is noted by improved growth, enzymatic activity, serum biochemical parameters, and immune functions.

### 4.2. Physiological Role of Manganese and Cobalt

For example, it is known that cobalt is used by ruminant rumen microorganisms to synthesize the tetrapyrrole ring known as corrin, vitamin B_12_ (C_63_H_88_O_14_N_14_PCo) [85, 86], and, consequently, cobalt is essential for corrin-dependent enzymes—organometallic cofactors, redox or adenosyl- and methylcobamides (ADOCBA, MECBA) [87], for example, methylmalonyl-CoA mutase, leucine mutase, and methionine synthetase, which catalyze the conversion of propionate to succinate, L-α-leucine to L-β-leucine, homocysteine to methionine, respectively, with simultaneous regeneration tetrahydrofolate, a precursor of purine and pyrimidine. However, the mobilization of cobalt by the microbiome from food substrates and inorganic salts is ineffective and amounts to about 13% under favorable conditions [88]. At the same time, Co-containing UFP, binding to proteins of the internal physiological environment, acquires a kind of bioidentity and is effectively sorbed by the epithelial lining of the gastrointestinal tract [89].

Similarly, manganese functions as a cofactor for arginase, glutamine synthetase, pyruvate carboxylase, Mn-SOD, and Mn-cofactored catalases and peroxidase, and is involved, respectively, in the hydrolysis of arginine to ornithine and urea, the ATP-dependent conversion of glutamate to glutamine, the synthesis of oxalacetate from pyruvate, and the neutralization of superoxide anion radicals [90]. It is also responsible for the metabolism of macronutrients, bone formation, ammonia clearance, and the synthesis of neurotransmitters in the brain. In addition, manganese has been shown to replace iron in some Fe-mononuclear enzymes in *Escherichia coli* under oxidative stress, protecting them from Fenton reaction–mediated damage [91]. However, as with cobalt, manganese sorption in the body of ruminants is extremely low, amounting to no more than 1% [92]. At the same time, the introduction of Mn-deposited UFP with explicit antibiotic properties against both gram-negative and gram-positive microorganisms, as well as against fungi, for example, *Trichophyton simii, Curvularia lunata, Aspergillus niger*, and *Candida albicans* [93] into the poultry diet significantly increased the absorption of this microelement in the ileum [94].

Accordingly, manganese and cobalt as components of metalloenzymes are critically important for a wide range of metabolic processes, including the metabolism of proteins, fats, carbohydrates and nucleic acids, growth and development, digestion and detoxification, energy production, and regulation of neuronal activity [88, 90]. The most promising form of their introduction into the diet of animals is UFP, which allows reducing the required dosages in comparison with bulk minerals and the excretion of elements with undigested feed residues against the background of increased bioavailability. As a result, the burden on the environment and economic costs is reduced [51].

### 4.3. Dynamics of Digestive Processes

The change in the dynamics of digestive processes in rumen in vitro with the addition of macro- and microelements in various forms including UFP can thus be due to two complementary mechanisms already mentioned earlier, namely, the incorporation of metal ions into physiological processes and their antibiotic effect against a number of opportunistic and some other commensal forms [32, 72, 93].

The latter, given the complexity and diversity of intrasystemic interactions of microbiota, includes integration between:

1. Fibrolytic and proteolytic bacteria (responsible for the elimination of protein breakdown products—branched-chain fatty acids and ammonia);
2. Succinate-producing and utilizing prokaryotes (determines the conversion of acetic acid into propionic acid);
3. Lactate-producing and lactate-degrading microorganisms (*Megasphaera elsdenii* and *Selenomonas ruminantium* convert lactic acid into acetate, propionate, and butyrate);
4. Interspecies hydrogen transfer (increasing the concentration of acetate and ATP while reducing the amount of reduced fermentation products, such as lactate, ethanol, succinate, and propionate).

Taken together, all of the above directly affect the general taxonomic profile and the overall metabolic effect [95].

Thus, in particular, feeding Kazakh White-headed bulls with CoCl_2_ in the amount of 39 mg/head, a shift toward gram-negative bacteria with a decrease in the proportion of gram-positive bacteria was observed in rumen contents; accordingly, the number of representatives of *Verrucomicrobia* and *Bacteroidetes* types increased [96]. At the same time, the total number of species decreased relative to the control group by 2.4%, and the Simpson index was 50% lower, which indicates a more uniform distribution of prokaryotes in the community. All this, in essence, allows us to talk about the possibilities of modulating the cellulolytic, amylolytic, saccharolytic, lipolytic, proteolytic, and fibrolytic activity of the rumen contents [95, 97, 98].

At the same time, it was established that an increase in the concentration of cobalt in the environment of propionic acid bacteria *Propionibacterium freudenreichii* stimulates the synthesis and accumulation of corrinoids [99]. They also intensify the reproduction of ciliates [100], in particular herbivorous representatives of the genera *Entodinium* and *Diplodinium* (*Entodinium nanellum, Entodinium ovinum, Diplodinium bubalidis ssp. bubalidis*), which have cellulolytic activity, as well as individual species that break down starch with the formation of acetic, propionic, and butyric acids—*Entodinium ecaudatum, Isotricha intestinalis, Dasytricha ruminantium, Entodinium simulans—dubardi*, *Ophryoscolex caudatus*. It corresponds to the previously described dynamics of enzymatic activity, total, and protein nitrogen. Moreover, most endobiont ciliates, and especially some predatory individuals (*Entodinium bursa*), actively eat bacteria, thereby restraining their mass reproduction [101].

As for manganese, it is especially necessary for representatives of the genera *Lactiplantibacillus* and *Lacticaseibacillus*, as well as, to a lesser extent, *Bacillus subtilis* and other *Bacillota (Firmicutes)* to neutralize ROS, regulate growth and development processes [102]. In turn, lactic acid bacteria and *Bacillus subtilis*, which produce antimicrobial peptides, bacteriocins, have pronounced probiotic characteristics: They modulate the microbiome; promote fiber digestibility; reduce methane emissions, the risk of acidosis and allergic reactions, and the excretion of *Escherichia coli* with feces; and increase the concentration of VFA and the productivity of ruminants [103–105]. By competitive exclusion in the process of symbiosis, they form the host's food immunity, since manganese is also associated with the virulence of some prokaryotes [102]. At the same time, supplementation of manganese sulfate and chelate with a basal content of the trace element of 150 mg/kg feed DM in lambs diet promoted the nutrient digestibility and increased the biomass of the protozoan and bacterial fraction of the rumen content [106]. Similarly, Mn-methionine, MnSO_4_, and MnCl_2_ increased VFA concentration, in particular acetate and propionate, ammonia nitrogen, DM digestibility, amylase, trypsin, cellulase and lipase activities, and the microbial protein content in yaks [107].

In other words, UFPs of essential elements have greater potential in animal husbandry as effectors of rumen digestion than inorganic salts. However, it should be noted that the overall effect after their introduction is determined not only by dosages and physicochemical characteristics, but also by complex interactions with other components of premixes and feed substrate [108, 109], which determines the relevance of further studies of the mechanisms of metabolic inclusions of UFPs.

## 5. Conclusions

Thus, UFPs Mn_2_O_3_ and Co_3_O_4_ demonstrated significant potential as effectors of digestive processes in the rumen, stimulating the reproduction of protozoa and the enzymatic activity of the microbiome, which in combination ensured an increase in the digestibility of DM of feed. In other words, they can be used in the future as feed additives for ruminants. However, to fully understand the mechanisms of their action, it is also necessary to analyze the microbiome and metabolic pathways in the rumen.

## Figures and Tables

**Table 1:** Dynamics of toxicity of UFP Mn_2_O_3_ in relation to the constitutively luminescent strain *Escherichia coli K12 TG1*.

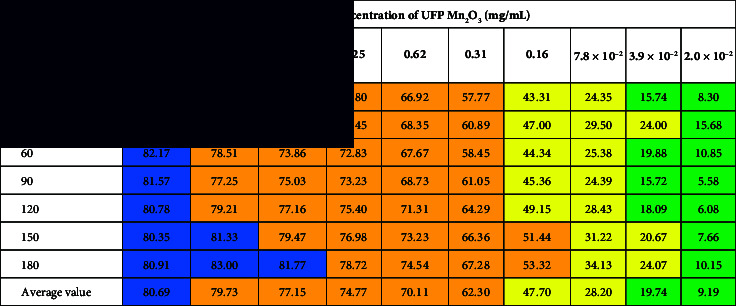

*Note:* Numerical values correspond to the toxicity index *T* (%). Color fill corresponds to indicators 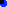
—ЕС_80_, 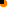
—ЕС_50_, 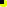
—ЕС_20_, 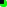
—NTOX, i.e., concentrations of UFP causing over 95%, 80%, 50%, and 20% quenching of the biosensor and nontoxic doses compared to the control.

**Table 2:** Toxicity dynamics of UFP Co_3_O_4_ in relation to the constitutively luminescent strain *Escherichia coli K12 TG1*.

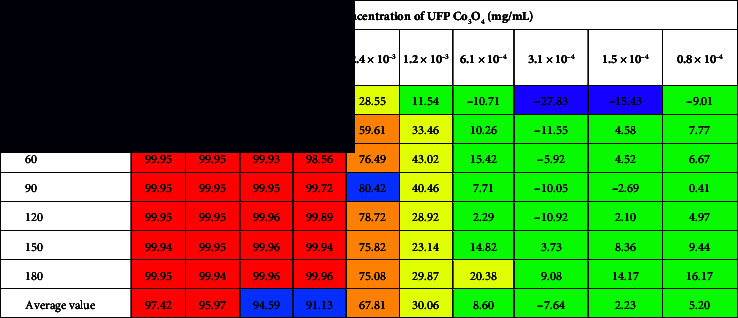

*Note:* Numerical values correspond to the toxicity index *T* (%). Color fill corresponds to indicators 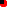
—Tox, 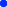
—ЕС_80_, 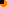
—ЕС_50_, 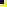
—ЕС_20_, 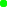
—NTOX, 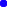
—NTOX^+^, i.e., concentrations of UFPs causing over 95%, 80%, 50%, and 20% quenching of the biosensor, as well as nontoxic and luminescence-stimulating (from −15% to −50% by toxicity index) compared to the control.

**Table 3:** Enzymatic activity of the microbiome upon addition of the studied UFPs, *n* = 5.

Enzymatic activity	Groups					
	Control	Mn_2_O_3_	P-level	Co_3_O_4_	P-level	P-level (Mn_2_O_3_ to Co_3_O_4_)
Cellulolytic activity (mg/h)	0.77 ± 0.02	0.91 ± 0.01	0.012	1.04 ± 0.02	0.012	0.012
Amylolytic activity (mg/h)	0.58 ± 0.03	3.57 ± 0.04	0.012	1.8 ± 0.05	0.012	0.012
Proteinase activity (μmol/h)	648.82 ± 7.08	598.48 ± 9.12	0.022	705.84 ± 7	0.012	0.012
Lipolytic activity (μmol/h)	12.4 ± 0.75	27.6 ± 1.17	0.012	21.6 ± 1.6	0.011	0.022

## Data Availability

The datasets generated during the current study are available from the corresponding author upon reasonable request.

## Supporting Information

Additional information contains the initial numerical data for figures and tables.

Additional material includes the initial laboratory data on the experiment, algorithms for calculating the average, standard deviation, Mann–Whitney coefficient, and Spearman correlation coefficient, as well as accompanying source graphs, diagrams, and tables. In the excel document «Initial data, figures and tables» on Sheet 1 are the results of assessing the biological activity, hydrodynamic diameter, and zeta potential of UFP Co_3_O_4_ and Mn_2_O_3_; on Sheets 2 and 3 are the initial data on these indicators; on Sheet 4 are graphs for assessing the effect of UFP on the coefficient of digestibility, the number of protozoa, and the microbial biomass of the rumen fluid; on Sheet 5 are the results of the assessment of the enzymatic activity of the rumen microbiota; on Sheet 6 are a graph for assessing the dynamics of production of various forms of nitrogen; and on Sheet 7 are statistical data processing.

## References

[1] Khan I., Saeed K., Khan I. (2019). Nanoparticles: Properties, Applications and Toxicities. *Arabian Journal of Chemistry*.

[2] Zhang L., Gu F. X., Chan J. M., Wang A. Z., Langer R. S., Farokhzad O. C. (2008). Nanoparticles in Medicine: Therapeutic Applications and Developments. *Clinical Pharmacology & Therapeutics*.

[3] Bai D. P., Lin X. Y., Huang Y. F., Zhang X. F. (2018). Theranostics Aspects of Various Nanoparticles in Veterinary Medicine. *International Journal of Molecular Sciences*.

[4] Pestovsky Y. S., Martínez-Antonio A. (2017). The Use of Nanoparticles and Nanoformulations in Agriculture. *Journal of Nanoscience and Nanotechnology*.

[5] Wang K., Lu X., Lu Y. (2022). Nanomaterials in Animal Husbandry: Research and Prospects. *Frontiers in Genetics*.

[6] Li X., Li W., Wang M., Liao Z. (2021). Magnetic Nanoparticles for Cancer Theranostics: Advances and Prospects. *Journal of Controlled Release*.

[7] Tang K. S. (2019). The Current and Future Perspectives of Zinc Oxide Nanoparticles in the Treatment of Diabetes Mellitus. *Life Sciences*.

[8] Babaie S., Taghvimi A., Hong J. H., Hamishehkar H., An S., Kim K. H. (2022). Recent Advances in Pain Management Based on Nanoparticle Technologies. *Journal of Nanobiotechnology*.

[9] Kan S., Hariyadi D. M., Grainge C., Knight D. A., Bartlett N. W., Liang M. (2020). Airway epithelial-targeted Nanoparticles for Asthma Therapy. *American Journal of Physiology: Lung Cellular and Molecular Physiology*.

[10] Gamazo C., Gastaminza G., Ferrer M., Sanz M. L., Irache J. M. (2014). Nanoparticle based-immunotherapy Against Allergy. *Immunotherapy*.

[11] Fathi-Achachelouei M., Knopf-Marques H., Ribeiro da Silva C. E. (2019). Use of Nanoparticles in Tissue Engineering and Regenerative Medicine. *Frontiers in Bioengineering and Biotechnology*.

[12] Raghunath A., Perumal E. (2017). Metal Oxide Nanoparticles as Antimicrobial Agents: a Promise for the Future. *International Journal of Antimicrobial Agents*.

[13] Chen L., Liang J. (2020). An Overview of Functional Nanoparticles as Novel Emerging Antiviral Therapeutic Agents. *Materials Science and Engineering: C*.

[14] Dadwal A., Baldi A., Kumar Narang R. (2018). Nanoparticles as Carriers for Drug Delivery in Cancer. *Artificial Cells, Nanomedicine, and Biotechnology*.

[15] Ivanishcheva A. P., Sizova E. A. (2021). The environmental-biology Aspects of Use of Chitosan and Ultrafine Particles of Copper and Iron in the Nutrition of Broiler Chickens. *IOP Conference Series: Earth and Environmental Science*.

[16] Kaningini A. G., Nelwamondo A. M., Azizi S., Maaza M., Mohale K. C. (2022). Metal Nanoparticles in Agriculture: a Review of Possible Use. *Coatings*.

[17] Rehmanullah M., Z., Muhammad Z., Inayat N., Majeed A. (2020). Application of Nanoparticles in Agriculture as Fertilizers and Pesticides: Challenges and Opportunities. *New frontiers in stress management for durable agriculture*.

[18] Amna, Alharby H. F., Hakeem K. R., Qureshi M. I. (2019). Weed Control Through herbicide-loaded Nanoparticles. *Nanomaterials and plant potential*.

[19] Pérez-Etayo L., González D., Leiva J. (2021). Antibacterial Activity of kaolin–silver Nanomaterials: Alternative Approach to the Use of Antibiotics in Animal Production. *Antibiotics*.

[20] Cheng G., Hao H., Xie S. (2014). Antibiotic Alternatives: the Substitution of Antibiotics in Animal Husbandry?. *Frontiers in Microbiology*.

[21] Skandalis N., Maeusli M., Papafotis D. (2021). Environmental Spread of Antibiotic Resistance. *Antibiotics*.

[22] Singer R. S., Finch R., Wegener H. C., Bywater R., Walters J., Lipsitch M. (2003). Antibiotic Resistance—The Interplay Between Antibiotic Use in Animals and Human Beings. *The Lancet Infectious Diseases*.

[23] Perreten V., Schwarz F., Cresta L., Boeglin M., Dasen G., Teuber M. (1997). Antibiotic Resistance Spread in Food. *Nature*.

[24] Kumar S. B., Arnipalli S. R., Ziouzenkova O. (2020). Antibiotics in Food Chain: the Consequences for Antibiotic Resistance. *Antibiotics*.

[25] Gopi M., Pearlin B., Kumar R. D., Shanmathy M., Prabakar G. (2017). Role of Nanoparticles in Animal and Poultry Nutrition: Modes of Action and Applications in Formulating Feed Additives and Food Processing. *International Journal of Pharmacology*.

[26] Chen J., Wang W., Wang Z. (2011). Effect of nano-zinc Oxide Supplementation on Rumen Fermentation in Vitro. *Chinese Journal of Animal Nutrition*.

[27] Riazi H., Rezaei J., Rouzbehan Y. (2019). Effects of Supplementary nano-ZnO on in Vitro Ruminal Fermentation, Methane Release, Antioxidants, and Microbial Biomass. *Turkish Journal of Veterinary and Animal Sciences*.

[28] Hosseini-Vardanjani S. F., Rezaei J., Karimi-Dehkordi S., Rouzbehan Y. (2020). Effect of Feeding nano-ZnO on Performance, Rumen Fermentation, Leukocytes, Antioxidant Capacity, Blood Serum Enzymes and Minerals of Ewes. *Small Ruminant Research*.

[29] Rajendran D., Kumar G., Ramakrishnan S., Shibi T. K. (2013). Enhancing the Milk Production and Immunity in Holstein Friesian Crossbred Cow by Supplementing Novel Nano Zinc Oxide. *Research Journal of Biotechnology*.

[30] Abedini M., Shariatmadari F., Karimi Torshizi M. A., Ahmadi H. (2018). Effects of Zinc Oxide Nanoparticles on the Egg Quality, Immune Response, Zinc Retention, and Blood Parameters of Laying Hens in the Late Phase of Production. *Journal of Animal Physiology and Animal Nutrition*.

[31] Shi L., Xun W., Yue W. (2011). Effect of Sodium Selenite, Se-yeast and nano-elemental Selenium on Growth Performance, Se Concentration and Antioxidant Status in Growing Male Goats. *Small Ruminant Research*.

[32] Moradpoor H., Safaei M., Rezaei F. (2019). Optimisation of Cobalt Oxide Nanoparticles Synthesis as Bactericidal Agents. *Open access Macedonian journal of medical sciences*.

[33] Shi L., Xun W., Yue W. (2011). Effect of Elemental nano-selenium on Feed Digestibility, Rumen Fermentation, and Purine Derivatives in Sheep. *Animal Feed Science and Technology*.

[34] Shi L. G., Yang R. J., Yue W. B. (2010). Effect of Elemental nano-selenium on Semen Quality, Glutathione Peroxidase Activity, and Testis Ultrastructure in Male Boer Goats. *Animal Reproduction Science*.

[35] Mohamed M. Y., Ibrahim E. M. M., Abd El-Mola A. (2017). Effect of Selenium Yeast And/or Vitamin E Supplemented Rations on Some Physiological Responses of Post-lambing Ossimi Ewes Under Two Different Housing Systems. *Egyptian Journal of Nutrition and Feeds*.

[36] Gangadoo S., Dinev I., Chapman J. (2018). Selenium Nanoparticles in Poultry Feed Modify Gut Microbiota and Increase Abundance of Faecalibacterium prausnitzii. *Applied Microbiology and Biotechnology*.

[37] Stepanova I. A., Nazarova A. A., Arisov M. V. (2020). Peculiarities of Mineral Metabolism of Holstein Heifers’ Diet Supplemented with Copper Nanopowders. *World’s Veterinary Journal*.

[38] Byshova D. N., Ampleeva L. E., Polischuk S. D. (2022). Influence of Metal Nanopowders on Physiological Indices of black-and-white Calves. *Proceedings of the XII International Youth Scientific Conference “Youth and the XXI Century*.

[39] kazaz S., Hafez M. H. (2020). Evaluation of Copper Nanoparticles and Copper Sulfate Effect on Immune Status, Behavior, and Productive Performance of Broilers. *Journal of Advanced Veterinary and Animal Research*.

[40] Gonzales-Eguia A., Fu C. M., Lu F. Y., Lien T. F. (2009). Effects of Nanocopper on Copper Availability and Nutrients Digestibility, Growth Performance and Serum Traits of Piglets. *Livestock Science*.

[41] Wang M. Q., Xu Z. R., Zha L. Y., Lindemann M. D. (2007). Effects of Chromium Nanocomposite Supplementation on Blood Metabolites, Endocrine Parameters and Immune Traits in Finishing Pigs. *Animal Feed Science and Technology*.

[42] Wang M. Q., Xu Z. R. (2004). Effect of Chromium Nanoparticle on Growth Performance, Carcass Characteristics, Pork Quality and Tissue Chromium in Finishing Pigs. *Asian-Australasian Journal of Animal Sciences*.

[43] Fondevila M., Herrer R., Casallas M. C., Abecia L., Ducha J. J. (2009). Silver Nanoparticles as a Potential Antimicrobial Additive for Weaned Pigs. *Animal Feed Science and Technology*.

[44] Czyż K., Dobrzański Z., Wyrostek A., Senze M., Kowalska-Góralska M., Janczak M. (2023). The Effect of Nanosilver-based Preparation Added to Litter on Silver and Antagonistic Elements Content in Broiler Tissues and Organs. *Agriculture*.

[45] Ahmadi F., Branch S. (2012). Impact of Different Levels of Silver Nanoparticles (Ag-NPs) on Performance, Oxidative Enzymes and Blood Parameters in Broiler Chicks. *Pakistan Veterinary Journal*.

[46] Ahmadi F., Kurdestany A. H. (2010). The Impact of Silver Nano Particles on Growth Performance, Lymphoid Organs and Oxidative Stress Indicators in Broiler Chicks. *Global Veterinaria*.

[47] Andi M. A., Hashemi M., Ahmadi F. (2011). Effects of Feed Type with/without Nanosil on Cumulative Performance, Relative Organ Weight and Some Blood Parameters of Broilers. *Global Veterinaria*.

[48] Rajendran D. (2013). Application of Nano Minerals in Animal Production System. *Research Journal of Biotechnology*.

[49] Kvan O. V., Sizova E. A., Kamirova A. M. (2021). Microbial Biodiversity of Cecum in Broiler Chickens when Adding Various Ultrafine Particles to Feed. *Ministry of Agriculture of Russian Federation*.

[50] Patra A., Lalhriatpuii M. (2020). Progress and Prospect of Essential Mineral Nanoparticles in Poultry Nutrition and Feeding—A Review. *Biological Trace Element Research*.

[51] Michalak I., Dziergowska K., Alagawany M. (2022). The Effect of metal-containing Nanoparticles on the Health, Performance and Production of Livestock Animals and Poultry. *Veterinary Quarterly*.

[52] Ezzat Abd M., Alagawany M., Ragab Fara M. (2017). Nutritional and Pharmaceutical Applications of Nanotechnology: Trends and Advances. *International Journal of Pharmacology*.

[53] Pajerski W., Ochonska D., Brzychczy-Wloch M. (2019). Attachment Efficiency of Gold Nanoparticles by Gram-positive and Gram-negative Bacterial Strains Governed by Surface Charges. *Journal of Nanoparticle Research*.

[54] Lynch I., Cedervall T., Lundqvist M., Cabaleiro-Lago C., Linse S., Dawson K. A. (2007). The nanoparticle-protein Complex as a Biological Entity; a Complex Fluids and Surface Science Challenge for the 21st Century. *Advances in Colloid and Interface Science*.

[55] Abdelnour S. A., Alagawany M., Hashem N. M. (2021). Nanominerals: Fabrication Methods, Benefits and Hazards, and Their Applications in Ruminants with Special Reference to Selenium and Zinc Nanoparticles. *Animals*.

[56] Khalili Fard J., Jafari S., Eghbal M. A. (2015). A Review of Molecular Mechanisms Involved in Toxicity of Nanoparticles. *Advanced Pharmaceutical Bulletin*.

[57] Zoroddu M. A., Medici S., Ledda A., Nurchi V. M., Lachowicz J. I., Peana M. (2014). Toxicity of Nanoparticles. *Current Medicinal Chemistry*.

[58] Jamkhande P. G., Ghule N. W., Bamer A. H., Kalaskar M. G. (2019). Metal Nanoparticles Synthesis: an Overview on Methods of Preparation, Advantages and Disadvantages, and Applications. *Journal of Drug Delivery Science and Technology*.

[59] Shemetov A. A., Nabiev I., Sukhanova A. (2012). Molecular Interaction of Proteins and Peptides with Nanoparticles. *ACS Nano*.

[60] Mu Q., Jiang G., Chen L. (2014). Chemical Basis of Interactions Between Engineered Nanoparticles and Biological Systems. *Chemical Reviews*.

[61] Katsnelson B. A., Privalova L. I., Gurvich V. B. (2013). Comparative *in Vivo* Assessment of Some Adverse Bioeffects of Equidimensional Gold and Silver Nanoparticles and the Attenuation of Nanosilver’s Effects with a Complex of Innocuous Bioprotectors. *International Journal of Molecular Sciences*.

[62] Parvez S., Venkataraman C., Mukherji S. (2006). A Review on Advantages of Implementing Luminescence Inhibition Test (*Vibrio fischeri*) for Acute Toxicity Prediction of Chemicals. *Environment International*.

[63] Sizova E., Miroshnikov S., Yausheva E., Kosyan D. (2015). Comparative Characteristic of Toxicity of Nanoparticles Using the Test of Bacterial Bioluminescence. *Biosciences Biotechnology Research Asia*.

[64] Sizova E. A., Miroshnikov S. A. (2016). Bioecological Assessment of Various Test Objects in Contact with Metals in Nanoform. *Actual biotechnology*.

[65] Zewdie A. K. (2019). The Different Methods of Measuring Feed Digestibility: a Review. *EC Nutr*.

[66] Senko A. V., Voronov D. V. (2010). Methodological Recommendations for the Study of the Contents of the Rumen in Cows. *VKN: Grodno State Agrarian University*.

[67] Cypriyana P J J., Saigeetha S., Angalene J L. A. (2021). Overview on Toxicity of Nanoparticles, It's Mechanism, Models Used in Toxicity Studies and Disposal methods–A Review. *Biocatalysis and Agricultural Biotechnology*.

[68] Wang H., Ren T., Zhu N., Yu Q., Li M. (2018). Co_3_O_4_ Nanoparticles at Sublethal Concentrations Inhibit Cell Growth by Impairing Mitochondrial Function. *Biochemical and Biophysical Research Communications*.

[69] Pogribna M., Koonce N. A., Mathew A. (2020). Effect of Titanium Dioxide Nanoparticles on DNA Methylation in Multiple Human Cell Lines. *Nanotoxicology*.

[70] Kolesnikov S. I., Varduni V. M., Timoshenko A. N. (2020). Assessment of Ecotoxicity of Cobalt, Copper, Nickel and Zinc Oxide Nanoparticles Based on Biological Indicators of Ordinary Chernozem Condition. *South of Russia: Ecology, Development*.

[71] Sharan A., Nara S. (2020). Exposure of Synthesized Co_3_O_4_ Nanoparticles to *Chlorella Minutissima*: an Ecotoxic Evaluation in Freshwater Microalgae. *Aquatic Toxicology*.

[72] Gupta V., Kant V., Sharma A. K., Sharma M. (2020). Comparative Assessment of Antibacterial Efficacy for Cobalt Nanoparticles, Bulk Cobalt and Standard Antibiotics: a Concentration Dependant Study. *Nanosystems: Physics, Chemistry, Mathematics*.

[73] Otero-González L., García-Saucedo C., Field J. A., Sierra-Álvarez R. (2013). Toxicity of TiO_2_, ZrO_2_, Fe^0^, Fe_2_O_3_, and Mn_2_O_3_ Nanoparticles to the Yeast, *Saccharomyces cerevisiae*. *Chemosphere*.

[74] Hassan M. S., Amna T., Pandeya D. R. (2012). Controlled Synthesis of Mn_2_O_3_ Nanowires by Hydrothermal Method and Their Bactericidal and Cytotoxic Impact: a Promising Future Material. *Applied Microbiology and Biotechnology*.

[75] Taghavi Fardood S., Moradnia F., Yekke Zare F. (2024). Green Synthesis and Characterization of α-Mn_2_O_3_ Nanoparticles for Antibacterial Activity and Efficient visible-light Photocatalysis. *Scientific Reports*.

[76] Azhir E., Etefagh R., Mashreghi M., Pordeli P. (2015). Preparation, Characterization and Antibacterial Activity of Manganese Oxide Nanoparticles. *Physical Chemistry Research*.

[77] Hoseinzadeh E., Makhdoumi P., Taha P. (2017). A Review on nano-antimicrobials: Metal Nanoparticles, Methods and Mechanisms. *Current Drug Metabolism*.

[78] Rana S., Kumar A. (2022). Toxicity of Nanoparticles to algae-bacterial co-culture: Knowns and Unknowns. *Algal Research*.

[79] Kaweeteerawat C., Ivask A., Liu R. (2015). Toxicity of Metal Oxide Nanoparticles in *Escherichia coli* Correlates with Conduction Band and Hydration Energies. *Environmental Science and Technology*.

[80] Osman D., Cooke A., Young T. R., Deery E., Robinson N. J., Warren M. J. (2021). The Requirement for Cobalt in Vitamin B12: a Paradigm for Protein Metalation. *Biochimica et Biophysica Acta, Molecular Cell Research*.

[81] Mondal A. H., Behera T., Swain P. (2020). Nano Zinc Vis‐À‐Vis Inorganic Zinc as Feed Additives: Effects on Growth, Activity of Hepatic Enzymes and Non‐Specific Immunity in Rohu, Labeo Rohita (Hamilton) Fingerlings. *Aquaculture Nutrition*.

[82] Sawosz E., Łukasiewicz M., Łozicki A. (2018). Effect of Copper Nanoparticles on the Mineral Content of Tissues and Droppings, and Growth of Chickens. *Archives of Animal Nutrition*.

[83] Afshari A., Sourinejad I., Gharaei A., Johari S. A., Ghasemi Z. (2021). The Effects of Diet Supplementation with Inorganic and Nanoparticulate Iron and Copper on Growth Performance, Blood Biochemical Parameters, Antioxidant Response and Immune Function of Snow Trout Schizothorax zarudnyi (Nikolskii, 1897). *Aquaculture*.

[84] Makarov P. M., Stepanova I. A., Nazarova A. A., Polishchuk S. D., Churilov G. I. (2017). Physiological and Biochemical Parameters of Holstein Heifers when Adding to Their Diet bio-drugs Containing Cuprum and Cobalt Nanoparticles. *Nano Hybrids and Composites*.

[85] Warren M. J., Raux E., Schubert H. L., Escalante-Semerena J. C. (2002). The Biosynthesis of Adenosylcobalamin (Vitamin B12). *Natural Product Reports*.

[86] Hawco N. J., McIlvin M. M., Bundy R. M. (2020). Minimal Cobalt Metabolism in the Marine Cyanobacterium Prochlorococcus. *Proceedings of the National Academy of Sciences*.

[87] Kräutler B., Jaun B. M. (2022). Vitamin B 12 and Cofactor F430. *Fundamentals of Porphyrin Chemistry: A 21st Century Approach*.

[88] González-Montaña J. R., Escalera-Valente F., Alonso A. J., Lomillos J. M., Robles R., Alonso M. E. (2020). Relationship Between Vitamin B12 and Cobalt Metabolism in Domestic Ruminant: an Update. *Animals*.

[89] Carrillo-Carrion C., Carril M., Parak W. J. (2017). Techniques for the Experimental Investigation of the Protein Corona. *Current Opinion in Biotechnology*.

[90] Avila D. S., Puntel R. L., Aschner M. (2013). Manganese in Health and Disease. *Metal Ions in Life Sciences*.

[91] Sobota J. M., Imlay J. A. (2011). Iron Enzyme ribulose-5-phosphate 3-epimerase in *Escherichia coli* Is Rapidly Damaged by Hydrogen Peroxide but Can Be Protected by Manganese. *Proceedings of the National Academy of Sciences*.

[92] Spears J. W. (2019). Boron, Chromium, Manganese, and Nickel in Agricultural Animal Production. *Biological Trace Element Research*.

[93] Hoseinpour V., Ghaemi N. (2018). Green Synthesis of Manganese Nanoparticles: Applications and Future perspective–A Review. *Journal of Photochemistry and Photobiology B: Biology*.

[94] Matuszewski A., Łukasiewicz M., Łozicki A. (2020). The Effect of Manganese Oxide Nanoparticles on Chicken Growth and Manganese Content in Excreta. *Animal Feed Science and Technology*.

[95] Nagaraja T. G. (2016). Microbiology of the Rumen. *Rumenology*.

[96] Ryazanov V., Tarasova E., Duskaev G., Kolpakov V., Miroshnikov I. (2023). Changes in the Concentration of Amino Acids and Bacterial Community in the Rumen when Feeding Artemisia absinthium and Cobalt Chloride. *Fermentation*.

[97] Miroshnikova M. S. (2020). The Main Representatives of the Rumen Microbiome (Review). *Animal Husbandry and Fodder Production*.

[98] Koloskova E. M., Ostrenko K. S., Ezersky V. A., Ovcharova A. N., Belova N. V. (2020). Study of the Rumen Microbiome in Sheep Using Molecular Genetic Methods (Review). *Problems of biology of productive animals*.

[99] Kamenskaya Yu.V. (2019). The Effect of Cobalt Salts on the Biosynthesis of Vitamin B12 by Propionic Acid Bacteria. *Science, Technology and Education*.

[100] Bonhomme A., Durand M., Quintana C., Halpern S. (1982). Influence Du Cobalt Et De La Vitamine B12 Sur La Croissance Et La Survie Des Ciliés Du Rumen *in Vitro*, En Fonction De La Population Bactérienne. *Reproduction, Nutrition, Developpement*.

[101] Chernaya L. V. (2016). Features of the Vital Activity of Endobiont Ciliates in the Stomach of Sheep. *International Journal of Applied and Basic Research*.

[102] Bosma E. F., Rau M. H., van Gijtenbeek L. A., Siedler S. (2021). Regulation and Distinct Physiological Roles of Manganese in Bacteria. *FEMS Microbiology Reviews*.

[103] Bidarkar V. K., Swain P. S., Ray S., Dominic G. (2014). Probiotics: Potential Alternative to Antibiotics in Ruminant Feeding. *Trends in Veterinary and Animal Sciences*.

[104] Chang M., Ma F., Wei J., Liu J., Nan X., Sun P. (2021). Live Bacillus subtilis Natto Promotes Rumen Fermentation by Modulating Rumen Microbiota *in Vitro*. *Animals*.

[105] Doyle N., Mbandlwa P., Kelly W. J. (2019). Use of Lactic Acid Bacteria to Reduce Methane Production in Ruminants, a Critical Review. *Frontiers in Microbiology*.

[106] Gresakova L., Venglovska K., Cobanova K. (2018). Nutrient Digestibility in Lambs Supplemented with Different Dietary Manganese Sources. *Livestock Science*.

[107] Lu H., Liu P., Liu S. (2023). Effects of Sources and Levels of Dietary Supplementary Manganese on Growing Yak’s *in Vitro* Rumen Fermentation. *Frontiers in Veterinary Science*.

[108] Ilyichev E., Nazarova A., Polischuk S., Inozemtsev V. (2011). Diet Digestibility and Nutrient Balance After Feeding Calves with Cobalt and Copper Nanopowders. *Dairy and Beef Cattle Breeding*.

[109] Nurzhanov B. S., Levakhin Yu.I., Duskaev G. K., Zhaimysheva S. S. (2020). The Effect of *Cucurbita Esemenisoleum* Enriched with Highly Dispersed Manganese Particles on Dry Matter Digestibility and Microbiological Processes in the Rumen of Animals. *Bulletin of the Kurgan State Agricultural Academy*.

